# An intraspecific appraisal of the social intelligence hypothesis

**DOI:** 10.1098/rstb.2017.0288

**Published:** 2018-08-13

**Authors:** Benjamin J. Ashton, Alex Thornton, Amanda R. Ridley

**Affiliations:** 1 Centre for Evolutionary Biology, University of Western Australia, Western Australia 6009, Australia; 2 Centre for Ecology and Conservation, University of Exeter, Penryn Campus, Exeter TR10 9FE, UK

**Keywords:** social intelligence hypothesis, individual variation, Australian magpies, cognition, intraspecific

## Abstract

The prevailing hypotheses for the evolution of cognition focus on either the demands associated with group living (the social intelligence hypothesis (SIH)) or ecological challenges such as finding food. Comparative studies testing these hypotheses have generated highly conflicting results; consequently, our understanding of the drivers of cognitive evolution remains limited. To understand how selection shapes cognition, research must incorporate an intraspecific approach, focusing on the causes and consequences of individual variation in cognition. Here, we review the findings of recent intraspecific cognitive research to investigate the predictions of the SIH. Extensive evidence from our own research on Australian magpies (*Cracticus tibicen dorsalis*), and a number of other taxa, suggests that individuals in larger social groups exhibit elevated cognitive performance and, in some cases, elevated reproductive fitness. Not only do these findings demonstrate how the social environment has the potential to shape cognitive evolution, but crucially, they demonstrate the importance of considering both genetic *and* developmental factors when attempting to explain the causes of cognitive variation.

This article is part of the theme issue ‘Causes and consequences of individual differences in cognitive abilities’.

## 1. Introduction: prevailing theories for the evolution of cognition

For over a century, scientists have investigated the factors governing cognitive evolution, yet the topic still remains intensely debated today. Hypotheses typically place the emphasis on either social or ecological challenges as predominant factors driving cognitive evolution, but studies addressing the potential role of these factors have produced highly conflicting results [1–4].

The cognitive buffer hypothesis (CBH), for instance, predicts that large brain size evolved to allow species to adjust their behaviour adaptively in response to variable environmental conditions [5]. Two environmental challenges in particular are hypothesized to be selective pressures influencing cognition: resource availability and seasonality [3]. If the availability of food is difficult to predict, then selection may favour the evolution of enhanced learning and memory to allow animals to maximize foraging intake [6]. For example, frugivorous spider monkeys (*Ateles geoffroyi*), whose primary food source is ephemeral and unpredictable, have larger relative brain size compared to the leaf-eating howler monkey (*Alouatta palliate*), whose food source is ubiquitous [7]. These findings are supported by a recent phylogenetic analysis by DeCasien *et al.* [2], which found a strong relationship between frugivory and brain size. Coupled with evidence of links between brain size and cognitive ability [8–12], this appears consistent with the CBH. Further support comes from behavioural studies. Field studies suggest that grey-cheeked mangabeys (*Lophocebus albigena*), for example, use integrated, episodic memory about the location and ripeness of fruit encountered on previous foraging trips and weather conditions over the intervening period when deciding whether to revisit particular fruiting trees [13]. Unpredictable resource availability may also favour more innovative individuals if novel foraging techniques allow the exploitation of a new food source. In this vein, there is evidence of a positive relationship between innovativeness and brain size in both primates and birds (reviewed in [14]). Larger-brained birds (relative to body mass) are also more successful than smaller-brained birds when establishing themselves in a novel environment [12].

Although not necessarily mutually exclusive from resource availability, seasonality has also been hypothesized to select for increased cognitive ability [15,16]. Seasonal changes in climatic conditions mean some species migrate while others endure the harsher environments [15,16]. Both scenarios may create situations that select for elevated cognitive performance [15,16]. Migratory birds have increased levels of long-term spatial memory compared to non-migratory birds [17–19] (although note Sayol *et al.* [3] found migratory birds have smaller relative brain size). Among non-migratory species, it has been discovered that subpopulations that cache food for the winter tend to have a larger hippocampus and elevated long-term spatial memory retention compared to non-caching subpopulations [20–22]. A comparative analysis of brain size in 1200 bird species found larger-brained birds were more likely to occur in areas with greater environmental variation, adding support to the idea of seasonality favouring increased information processing power [3]. However, a number of comparative studies suggest ecological factors alone cannot adequately explain interspecific differences in neuroanatomy and cognition; for example, Shultz & Dunbar [23] concluded that social factors were just as important as ecological factors in driving the evolution of ungulate brains.

The novel concept of social intelligence was first introduced over half a century ago in papers by Chance & Mead [24] and Jolly [25], although it is arguably Nick Humphrey's seminal paper, ‘The social function of intellect’ [26], that is recognized as giving rise to the social intelligence hypothesis (SIH) and the resulting research in this area. The SIH posits that group living can generate substantial challenges that favour selection for enhanced cognitive abilities [27]. Since the SIH was conceptualized, an abundance of literature has characterized some of the potential challenges of living in groups [28]. The need to maintain and coordinate multiple relationships, monitor other group members and recognize suitable cooperative partners are examples of factors unique to social animals that are hypothesized to be selective pressures requiring advanced cognition [28,29]. Byrne & Whiten [30] also highlighted the ‘Machiavellian’ nature of some animal societies, where the need to outwit others in competitive interactions may generate arms races of escalating cognitive abilities.

The majority of evidence supporting the SIH is derived from comparative studies on primates and birds [31,32], relating between-species or between-population differences in brain size or cognitive performance to differences in social organization or life history [1,32–36]. Several proxies of social complexity have been found to correlate with cognitive performance or measures of brain size or brain composition [1,32–34]. For instance, large brain size in birds has been linked to the establishment, maintenance and coordination of behaviour within long-term, monogamous pair bonds [32]. In anthropoid primates, positive correlations between neocortex size and group size are argued to stem from the greater need to remember, track and manage relationships in larger groups [1]. Brain size is also particularly large in species with low within-group kinship, where individuals must make regular strategic decisions to manage conflicts of interest [37]. In addition, comparative studies have also revealed links between social structure and performance in a number of cognitive tasks. For instance, primates experiencing fission–fusion dynamics outperform those with more stable groups in tests of inhibitory control [34], and the highly social pinyon jay (*Gymnorhinus cyanocephalus*) outperforms less social corvids in transitive inference and reversal learning tasks [33,35].

Nevertheless, a number of studies have reported findings inconsistent with the SIH (reviewed in Holekamp [38]). For example, Sayol *et al*.'s [3] comprehensive analysis found no relationship between mating system and brain size in birds, and one of the largest avian forebrains (relative to total brain size) is found in the non-social owl (*Athene noctua*) [39]. It is also worth noting that the majority of comparative studies investigating cognitive evolution use neuroanatomical measures as proxies for cognition. The relationship between cognition and neuroanatomy remains highly contentious [40]; for example, it has been argued that gross measures of brain size, or brain regions, do not explicitly quantify neural functioning, and therefore, more refined neurobiological measures are required, such as neuron counting. Conversely, there is also evidence for a link between brain size and cognitive performance within, as well as between species (e.g. [9,41]).

The conflicting evidence generated from comparative studies suggests the need for a novel approach to the study of cognitive evolution. Recent studies focusing on *individual variation* in cognition have produced exciting results (e.g. the role that cognition plays in mate choice [42]), indicating that an intraspecific approach to the study of cognitive evolution, may be a valuable addition to comparative studies as a means of furthering our understanding of cognitive evolution.

## 2. An intraspecific approach to the study of cognition

A focus on individual differences in cognitive performance *within* species allows the causes and consequences of variation in cognitive ability to be quantified [43–47]. This is in contrast with an interspecific approach, where variation in cognitive performance within species is often disregarded as ‘noise’ [44] and species-level estimates of cognitive performance or brain size are used. This is also true of explanatory terms in analyses; for example, comparative analyses of the relationship between group size and brain size typically use average group size per species, despite there often being considerable intraspecific variation in group size [45]. Moreover, while comparative analyses often attempt to control for ecological and phylogenetic confounds, these are difficult to remove altogether and analyses can yield very different results depending on which variables are included and how those variables are specified [48]. Thus, rather than focusing exclusively on species- or population-level averages, vital insights may be gained by focusing on the causes of individual variation and linking cognitive variation to fitness consequences.

## 3. Intraspecific evidence for the social intelligence hypothesis?

Over the past decade, studies investigating intraspecific variation in cognitive performance have started to accumulate evidence for profound effects of both ecological factors and social factors. For instance, several studies on birds and fish have shown cognitive differences between individuals exposed to different climatic variables, predation pressure and feeding regimes (see table 1 for studies investigating the effect of the non-social environment on cognition), while zoo and laboratory studies show that enrichment of the physical environment can promote cognitive performance (e.g. [65,66]). Intraspecific studies are also generating evidence consistent with the SIH, showing that social factors can influence cognitive development, and in some cases that this may have important fitness consequences as well (see table 2 for studies investigating the effect of the social environment on cognition).

**Table 1.** Studies investigating the relationship between the non-social environment and individual cognitive performance/neuroanatomy in non-human animals (note this is an illustrative sample of studies, not a comprehensive list).

study	study species	measure of cognition	environmental variable	effect of environmental variable on measure of cognition?	fitness consequences?
Pravosudov & Clayton [49]	black-capped chickadee, *Poecile atricapillus*	hippocampal volume and spatial memory	altitude	positive	high altitude more efficient at cache recovery
Roth & Pravosudov [22]	*P. atricapillus*	hippocampal volume and neuron number	altitude	positive	not tested
Chancellor *et al.* [50]	*P. atricapillus*	hippocampal neurogenesis	altitude	positive	not tested
Freas *et al.* [51]	mountain chickadee, *P. gambelli*	spatial memory, hippocampal volume and neuron number	altitude	positive	not tested
Roth *et al.* [52]	*P. atricapillus*	hippocampal volume	altitude	positive	not tested
Freas *et al.* [53]	*P. gambelli; P. atricapillus*	hippocampal neuron soma size (volume, neuron number, neuron soma area)	altitude	positive	not tested
Freas *et al.* [54]	*P. gambelli*	hippocampal neuron soma size, volume, neuron number	altitude	positive	not tested
Roth *et al.* [55]	*P. atricapillus*	hippocampal glial cells	altitude	positive	not tested
Croston *et al.* [20]	*P. gambelli*	spatial memory	altitude	positive	not tested
Kotrschal & Taborsky [56]	cichlid, *Simochromis pleurospilus*	associative learning	environmental unpredictability	positive	not tested
Tebbich & Teschke [57]	woodpecker finch, *Cactospiza pallida*	reversal learning	environmental unpredictability	positive	not tested
Odling-Smee *et al*. [58]	three-spine stickleback, *Gasterosteus aculeatus*	spatial learning	environmental complexity	positive	not tested
Spence *et al*. [59]	zebra fish, *Danio rerio*	maze learning	environmental complexity	positive	not tested
Brown & Braithwaite [60]	poeciliid, *Brachyraphis episcopi*	spatial learning	predation pressure	negative	not tested
Brydges *et al*. [61]	three-spine stickleback	spatial learning	predation pressure	negative	not tested
Burns & Rodd [62]	guppy, *Poecilia reticulata*	spatial memory, telencephalon size	predation pressure	no effect	not tested
Croston *et al*. [63]	*P. gambelli*	spatial memory, reversal learning	altitude	mixed (no effect and negative)	not tested
Hermer *et al*. [64]	great tit, *Parus major*	reversal learning	altitude	mixed (no effect and negative)	not tested

**Table 2.** Studies investigating the relationship between the social environment and individual cognitive performance/neuroanatomy in non-human animals (note this is an illustrative sample of studies, not a comprehensive list).

study	study species	measure of cognition	measure of sociality	longitudinal testing?	effect of sociality on measure of cognition?	fitness consequences?
Ashton *et al.* [67]	Australian magpie (*C. tibicen dorsalis*)	behavioural inhibition, associative learning, reversal learning, spatial memory	group size	repeated testing of juveniles at 100, 200 and 300 days post-fledging	positive	positive relationship between cognitive performance and female reproductive success
Sallet *et al.* [68]	rhesus macaque, *Macaca mulatta*	size of various brain regions	social network size	not tested	positive	positive relationship between brain size and social dominance
Fischer *et al.* [69]	cichlid, *Neolamprologus pulcher*	size of various brain regions	rearing group size	not at the individual level, but treatment groups reared in isolation for different lengths of time	positive	not tested
Gonda *et al*. [70]	nine-spined stickleback, *Pungitius pungitius*	size of various brain regions	group size	not tested	positive	not tested
Fowler *et al.* [71]	prairie vole, *Microtus ochrogaster*	size of various brain regions	isolation versus male exposure	not at the individual level, but treatment groups reared in isolation for different lengths of time	positive	not tested
Lipkind *et al.* [72]	zebra finch, *Taenogypia guttata*	neuron number	group size	not tested	positive	not tested
Gonda *et al.* [73]	common frog*, Rana temporaria*	size of various brain regions	tadpole density	not tested	positive	not tested
Trokovic *et al*. [74]	*R. temporaria*	optic tecta	tadpole density	carry over effect from tadpole to froglet	positive	not tested
Ott & Rogers [75]	desert locust, *Schistocerca gregaria*	size of various brain regions	solitary versus gregarious	not tested	positive	not tested
Branchi *et al.* [76]	house mouse, *Mus musculus*	nerve growth factor	communal nest versus standard nest	not tested	positive	not tested
Dalesman [77]	pond snail*, Lymnaea stagnalis*	long-term memory	group living versus isolation	not tested	positive	not tested
Arnold & Taborsky [78]	cichlid, *N. pulcher*	social competence	parents and helpers versus no adults	not tested	positive	not tested
Taborsky *et al.* [79]	cichlid, *N. pulcher*	social competence	reared with older versus reared with same age conspecifics	not tested	positive	not tested
Seid & Junge [80]	ant*, Camponotus floridanus*	mushroom bodies	isolation versus groups	not at the individual level, but treatment groups reared in isolation for different lengths	positive	not tested
Smith *et al.* [81]	sweat bee, *Megalopta genalis*	mushroom bodies	social reproductives versus solitary reproductives	not tested	positive	not tested
Ehmer *et al*. [82]	paper wasp, *Polistes dominulus*	size of various brain regions	single foundress versus multiple foundress	not tested	positive	not tested
Amitai *et al.* [83]	rat, *Rattus norvegicus*	reversal learning	isolation reared versus socially reared	not tested	positive	not tested
Bianchi *et al.* [84]	rat, *R. norvegicus*	novel object recognition task	isolation reared versus socially reared	not tested	positive	not tested
Lu *et al.* [85]	rat*, R. norvegicus*	learning and spatial memory	group reared versus isolation reared	not at the individual level, but treatment groups reared in isolation for different lengths.	positive	not tested
Wongwitdecha & Marsden [86]	rat, *R. norvegicus*	place learning, reversal learning	isolation reared versus group reared	not tested	negative	not tested
Frisone *et al.* [87]	rat, *R. norvegicus*	spatial memory	isolation reared versus group reared	tested as juveniles and adults	mixed (negative and positive)	not tested
Riley *et al.* [88]	tree skink, *Egernia striolata*	motor, discrimination, and reversal learning	isolation reared versus group reared	not tested	no effect	not tested

The majority of evidence supporting a social theory of intellect is derived from studies on humans (see Kwak *et al.* [89] and references therein). For example, social network size has been found to predict orbital prefrontal cortex size [90] and ventromedial prefrontal volume [91], and Kanai *et al.* [92] found that online (as well as real world) social networks predicted right superior temporal sulcus, left middle temporal gyrus and entorhinal cortex size.

A small but growing number of studies, encompassing a broad range of taxa, are accumulating evidence linking social factors with intraspecific cognitive variation in non-human animals. For example, Sallet *et al.* [68] found that social network size correlates with levels of grey matter in the brains of rhesus macaques (*Mucaca mulatta*), and social enrichment has a positive effect on neural development in prairie voles [71] (*Microtus ochrogaster*) and mice [76] (*Mus musculus*). There is also evidence for a relationship between social group size and the development of various brain regions in invertebrates [75,80–82], amphibians [73,74], fish [69,70] and birds [72]. Although these studies suggest the social environment may play an important role in cognitive evolution, none of them directly quantify cognitive traits, with the majority of studies using measures of brain size or structure as a proxy for cognitive abilities (e.g. [69]); see Healy & Rowe [93] for a critical review of correlational studies of brain size or structure.

Intraspecific evidence suggests there is a relationship between sociality and cognition (table 2). However, it is worth noting that a number of these studies [83,84] use social isolation as a treatment: as isolation is likely to be highly stressful for social animals, these results may reflect pathological impacts of developmental stress rather than the cognitive demands of group living. Furthermore, none of these studies were carried out on wild populations of animals (table 2). To quantify and analyse variation in cognitive traits in ecologically relevant contexts, particularly in larger animals whose natural conditions cannot be readily replicated in the laboratory, it is vital to carry out tests on wild populations of animals, as selective pressures may be substantially different in captive conditions compared to the wild [47]. In order to determine the potential for selection to act on cognitive traits, it is also vital to examine the fitness consequences of variation in cognition, something that has rarely been attempted (table 2). Although reliably quantifying cognitive traits and monitoring fitness, especially in the wild, presents a number of challenges, [43,45,47], it is crucial if we are to further our understanding of factors shaping cognitive evolution.

## 4. Social influences on cognitive development in Australian magpies

**Figure 1.**
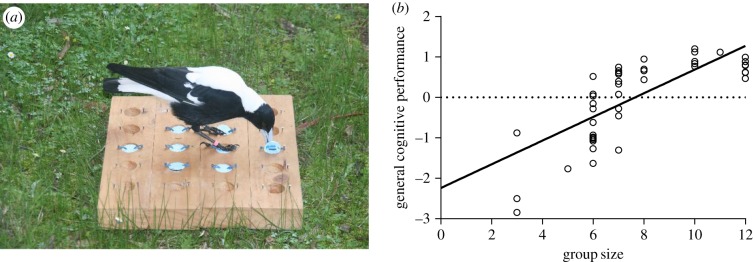
(*a*) An Australian magpie interacting with a cognitive task, and (*b*) the relationship between group size and general cognitive performance. Reproduced with permission from Ashton *et al.* [67]. (Online version in colour.)

We investigated the causes and consequences of individual variation in cognition in a wild population of Australian magpies (*Cracticus tibicen dorsalis*) at our field site in Perth, Western Australia. Cognitive performance was quantified by presenting individuals in 14 groups (ranging in size from three to 12 individuals) with a battery of four psychometric tests designed to measure inhibitory control, associative learning, reversal learning and spatial memory. We found individual performance was significantly positively correlated across all four tasks, and a principal component analysis (PCA) revealed evidence of a general cognitive factor (referred to as general cognitive performance hereafter) underlying cognitive performance [67]. Although there is evidence of general cognitive performance in a wide range of taxa [94–99], there are few examples of it being recorded in wild populations (although see [94,95]). Crucially, we found a strong positive association between group size and general cognitive performance ([67], figure 1). This relationship could not be explained by food intake (recorded during focal follows carried out outside of cognitive testing), body size, neophobia or time spent interacting with the task, suggesting that adults in large groups do well on tasks not because they are better fed or better able to focus on tasks, but rather because living in larger groups involves informational demands that affect cognitive development.

An important strength of the individual-based approach to the study of cognition is that it allows individual cognitive performance to be recorded over time [45]. Obtaining such longitudinal data allows us to determine factors affecting cognitive *development*. Few of the limited number of studies investigating the causes of intraspecific cognitive variation have attempted this (table 2), but there is growing evidence that the early social environment can affect brain development [69,74,76] and adult social learning ability [100]. In our research, we presented our cognitive test battery to juvenile Australian magpies at 100, 200 and 300 days post-fledging, finding that the relationship between group size and cognitive performance emerges as birds get older [67], adding to the weight of evidence that social factors can drive the development of domain-general cognitive abilities. One important, but as yet relatively unexplored issue, is whether this relationship may in fact be bi-directional: that is, while social factors may influence cognitive development, an individual's cognitive phenotype may also influence their social interactions with others [101].

## 5. Cognitive plasticity and evolution

Thus far, we have presented evidence that differences in the social environment experienced by different individuals may influence the development of their cognitive abilities. However, the SIH, as originally formulated, is an evolutionary, not a developmental hypothesis. To begin to address evolutionary questions, it is therefore necessary to ask whether elevated cognitive performance provides selective benefits. Previous studies using psychologically grounded psychometric tests have found both positive [102–106], negative [107] and no relationships [94,105] between individual cognitive performance and measures of fitness. In Australian magpies, we found that females that performed well in cognitive tasks had more successful nesting attempts, fledged more chicks and had more offspring that survived to independence [67]. Furthermore, cognitive performance was a stronger predictor of individual reproductive success than both foraging efficiency and body mass, indicating that variation in fitness was a direct consequence of cognitive performance, rather than nutritional intake [67]. Thus, it seems that in this species, the size of the group an individual grows up in influences the development of its cognitive performance, and cognitive performance in turn may have important consequences for reproductive fitness. As we were unable to manipulate group size experimentally, this proposed causal pathway cannot be demonstrated unequivocally, but these findings raise important questions. How does selection act upon a trait that is, at least in part, shaped by the social environment during early development? Is cognitive plasticity itself adaptive? Can developmental reaction norms themselves be shaped by selection? The answers to these questions have far reaching implications, not only in terms of understanding cognitive evolution, but also how we approach the study of evolution in general. Our results are consistent with the view that the proximate/ultimate distinction may be blurrier than is often suggested [108], as developmental processes may often be vital in shaping phenotypes that serve an ultimate function. Further work is needed to understand the interplay between development, inheritance and selection in shaping cognitive phenotypes.

## 6. Future directions

Although there is evidence for a link between sociality, cognition and fitness, the underlying mechanisms driving these associations are unclear. First, to unequivocally determine causality in the group size–cognition relationship, experimental manipulations of group size would be required. Cross-fostering experiments present the best opportunity to do this, but in the wild, they may only be feasible in species that breed synchronously and will accept eggs or young introduced from other groups. Another priority for future research is to determine precisely how and why group size affects cognitive development. Although we have argued that our findings cannot be explained by greater foraging intake for individuals in large groups, it is possible that nutrient quality, rather than total amount of food captured/received, drives cognitive development. Stable isotope analyses [109] may reveal if diet significantly differs between individuals from larger and smaller groups, and between individuals that exhibit differing cognitive performance. It is also important to characterize the social demands of living in larger groups, and relate these to cognitive development. Even within a group of a given size, different group members may well experience different information-processing demands, depending on the pattern of their agonistic and affiliative interactions and the strength and number of their relationships [110]. Social network analyses can help to quantify these relationships and characterize each individual's position within the wider social network (e.g. [111]), allowing us to relate each individual's cognitive profile to the specific social challenges it has faced during development.

A related point is the need to identify informational challenges more broadly. Studies of cognitive evolution have often tended to adopt a dichotomous approach: the key selection pressures acting on cognitive traits are *either* ecological *or* social. However, in reality, this distinction is not clear-cut: social animals, after all, solve ecological problems in a social context (see also [34]). Western-scrub jays (*Aphelocoma californica*), for example, use episodic memory to solve an ecological problem: remembering and retrieving food they have cached for the winter [112], but if there are other scrub jays present, they also face the need to outwit conspecifics so as to avoid having their caches stolen [113]. Thus, the problem is both ecological *and* social. Moreover, while some proponents of the SIH have argued that the demands of group living should specifically drive the evolution of *socio-cognitive* traits, there is increasing evidence that social behaviour often relies on the same, domain-general cognitive processes that are used to solve ecological problems [114,115]. Our research [67] speaks to this issue, in that social factors (specifically group size) appear to influence the development of basic cognitive processes (learning, memory and inhibitory control) that are not specifically social. The routes through which sociality affects cognitive development are still unknown, but could involve both explicitly social challenges (e.g. having to learn the characteristics of multiple different individuals) and ecological challenges that happen to be played out in a social context. For instance, if adults within a group show differing foraging niches [116], then dependent young in large groups may be exposed to a greater range of foraging locations, strategies and food types, driving elevated cognitive performance compared to youngsters in small groups. Thus, future studies may benefit from abandoning the explicit social/ecological dichotomy and focus instead on characterizing the full range of informational challenges that animals must solve.

Finally, while several studies have now identified a relationship between cognition and measures of fitness [102–106], our understanding of *why* cognition confers fitness benefits is limited. To resolve this, studies need to investigate how cognitive performance may influence the specific aspect of fitness being measured; for example, in the Australian magpie, an important next step will be to investigate if females with greater general cognitive performance provide offspring with improved parental care (perhaps through provisioning food of greater nutritional quality), and/or whether they are better at protecting their fledglings from threats from predators and conspecifics. Such research would have the potential to reveal *why* smarter females are capable of rearing offspring more successfully.

## 7. Conclusion

Understanding the factors driving cognitive evolution is one of the greatest challenges in biology today. Here, we highlight how several recent studies, using the relatively novel approach of focusing on the causes and consequences of individual variation in cognition, provide evidence of a link between sociality and cognition. While these results are broadly consistent with the SIH, we suggest that the distinction between social and ecological influences may, to a large extent, be artificial. Adopting an individual-based approach to the study of cognition will be important in revealing the information-processing challenges animals face in their physical and social environments, and elucidating the role of developmental processes in cognitive evolution.

## Data accessibility

This article has no additional data.
